# Supplementary material for: Quantifying factors that affect polygenic risk score performance across diverse ancestries and age groups for body mass index
Source: Pac Symp Biocomput. Author manuscript; Available in PMC 2023 Mar 16. (PMC10018532)

S Table 1. Effect of GWAS sample size on PRS performance. Difference in R^2 for different groupings of individuals between the UKBB GWAS down-sampled to that of the sample size used in GIANT (N=226,960), and the full UKBB European sample size (N=377,921). Test African ancestry individuals and 5,000 random European ancestry UKBB individuals were used as LD panels for all African and European ancestry test sets, respectively.

Age group	Ancestry	N	PRS R^2 UKBB Down-sampled sample size	PRS R^2 UKBB Full sample size	P-value
Adults	European	55418	0.0582	0.0734	$<4.94 \times 10^{-324}$
Teenagers		3114	0.0588	0.0667	3.75×10^{-9}
Children		5943	0.0274	0.0347	6.51×10^{-47}
Adults	African	5912	0.0140	0.0197	1.85×10^{-46}
Teenagers		1606	0.0179	0.0274	1.37×10^{-29}
Children		3668	0.00612	0.00715	4.55×10^{-9}

S Table 2. Selected Phecodes in eMERGE which were tested for their association to the PRS_{BMI}.

Phecode	Description	Cases/Controls (Adults)	
		EUR	AFR
401.1	Essential hypertension	38181/17166	4317/1588
272.1	Hyperlipidemia	32823/22524	2625/3280
272.11	Hypercholesterolemia	22537/32810	1912/3993
278.1	Obesity (positive control)	16348/38999	2282/3623
411.4	Coronary atherosclerosis	18548/36799	1449/4456
250.2	Type 2 diabetes	16342/39005	2525/3380
296.2	Depression	14651/40696	1630/4275
208	Benign neoplasm of colon	16254/39093	1205/4700
562.1	Diverticulosis	15656/39691	1068/4837
495	Asthma	9391/45956	1253/4652

S Figure 1. Differences in PRS R^2 across different age groups (the best performing LD panel was used for each run, p-values above each bar). We consistently observed more significant differences in PRS R^2 in children versus adults and teenagers. The down-sampled UKBB summary statistics were used for these comparisons.

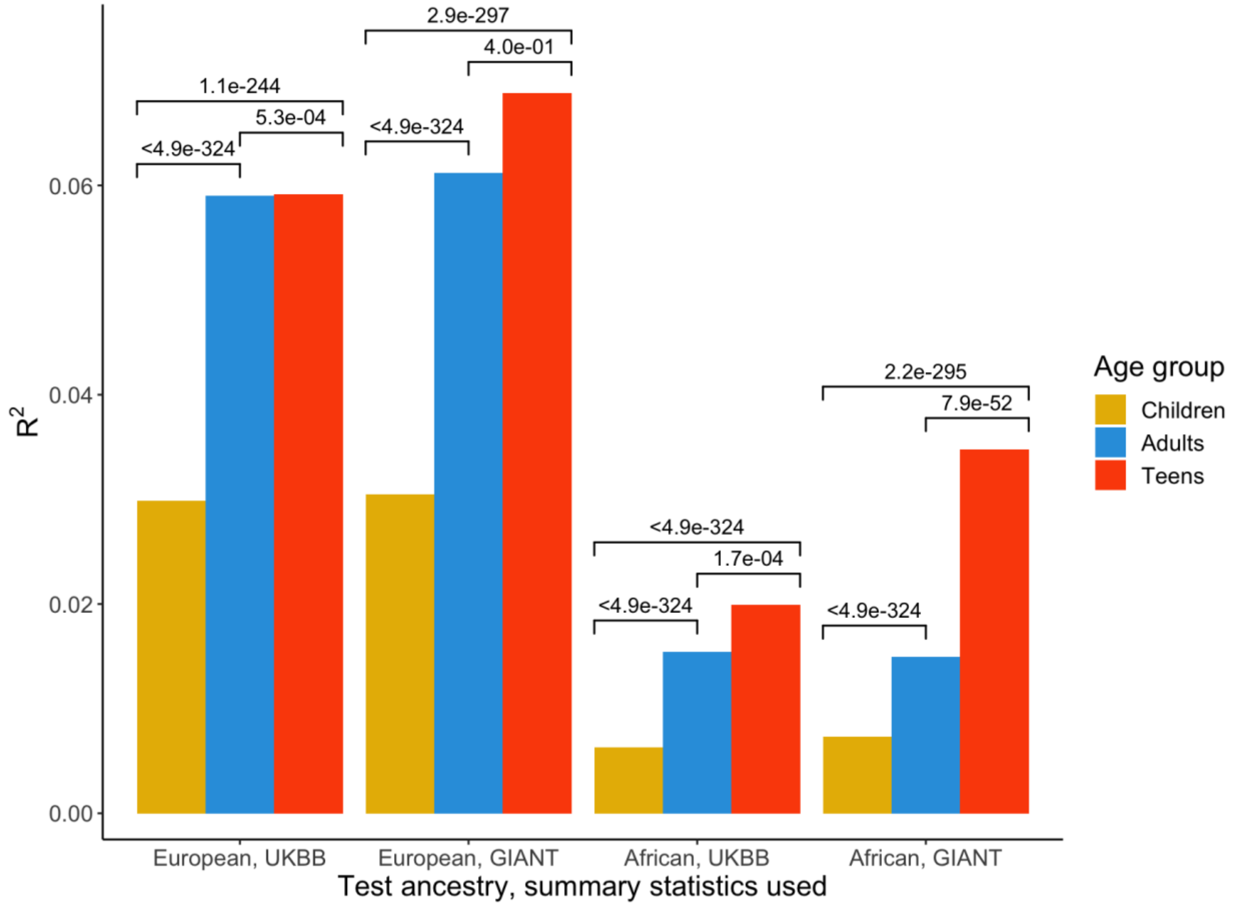

Supplement: Supplementary-9789811270611_0040 [file NIHMS1853022-supplement-Supplementary-9789811270611_0040.pdf]
